# Supplementary material for: Advanced Nanopharmaceutical Intervention for the Reduction of Inflammatory Responses and the Enhancement of Behavioral Outcomes in APP/PS1 Transgenic Mouse Models
Source: Pharmaceutics. 2025 Jan 31;17(2):177. doi: 10.3390/pharmaceutics17020177 (PMC11859494; doi:10.3390/pharmaceutics17020177)
Supplement: Supplementary file 1 [file pharmaceutics-17-00177-s001.zip › pharmaceutics-3421147-supplementary.pdf]

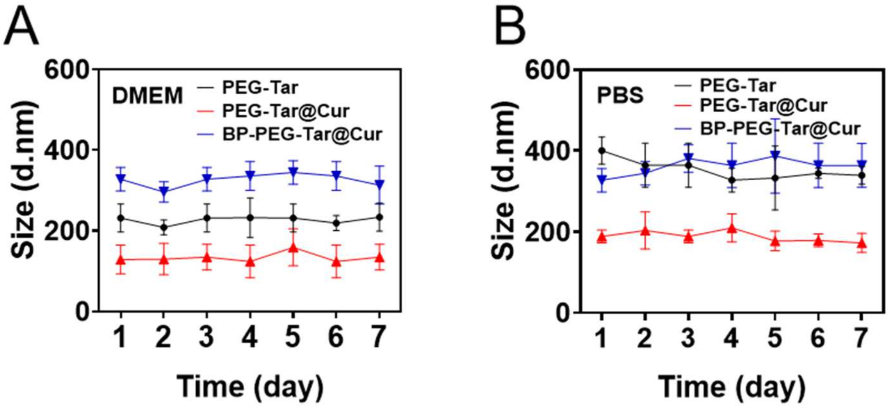

Figure S1. Size stability of various nanomaterials in two different solvents DMEM(A) and PBS(B).

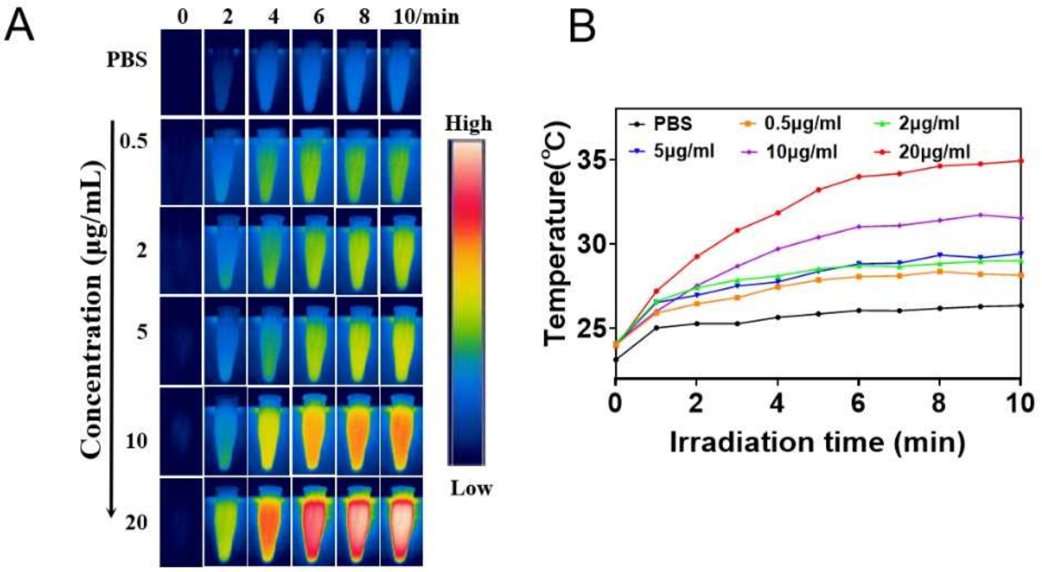

Figure S2. Temperature variation of different concentrations of BP-PEG-Tar@Cur solution and PBS under 808nm laser irradiation at a power density of 1W/cm^2. The thermal imaging pictures recorded during irradiation process (A). Specific temperature values during irradiation process (B).

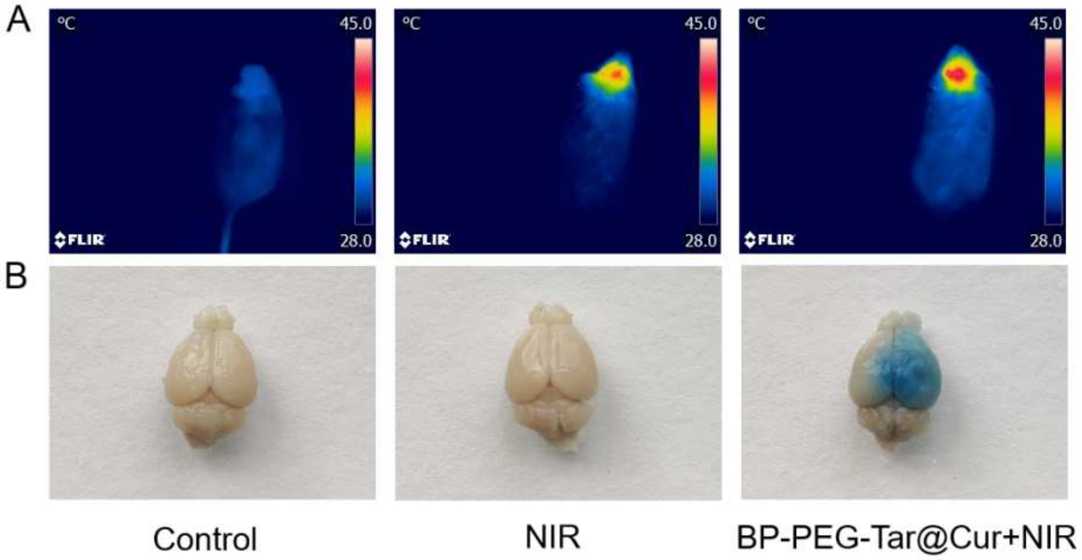

Figure S3. The results of the in vivo photothermal imaging experiment. (A) Thermal imaging maps of the mice from different groups. (B) Photographs of the extracted brains of mice corresponding to the thermal imaging maps.

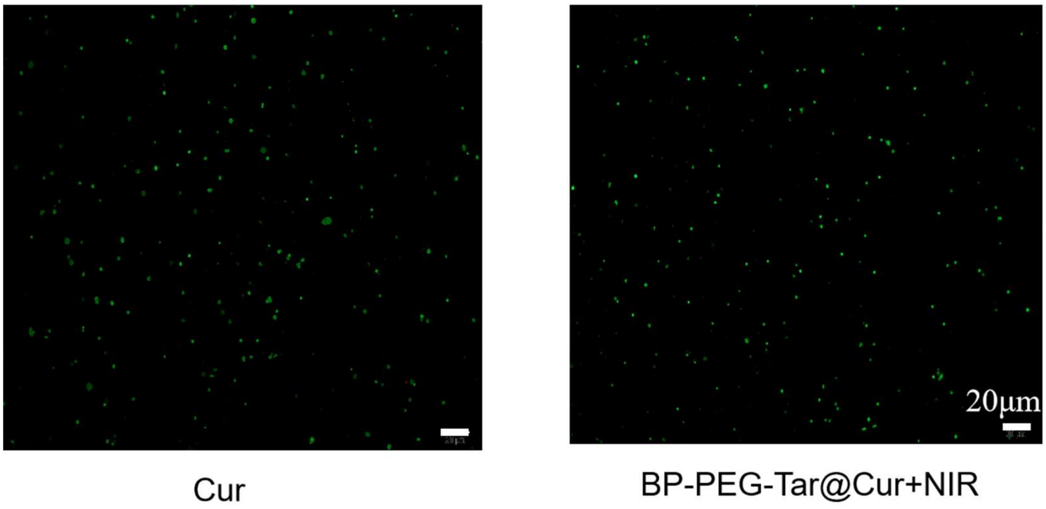

Figure S4. Fluorescence imaging results of cells in Transwell experiment treated with Cur and BP-PEG-Tar@Cur+NIR using Calcein-AM/PI staining method. (Scale bar: 20 μm)

Initial	Control	Cur	BP-PEG-Tar@Cur+NIR
resistance value (Ω)	110	153.6	158.3
TEER (Ω x cm ^{2}) = [(resistance in experimental hole- resistance blank hole)] x (4.67 cm 2) (membrane surface area)		203.6	225.6
**After irradiation**	**Control**	**Cur**	**BP-PEG-Tar@Cur+NIR**
resistance value (Ω)	109	153.3	155.6
TEER (Ω x cm ^{2}) = [(resistance in experimental hole- resistance blank hole)] x (4.67 cm 2) (membrane surface area)		206.9	217.6
**2 hour later**	**Control**	**Cur**	**BP-PEG-Tar@Cur+NIR**
resistance value (Ω)	107.6	154.7	160
TEER (Ω x cm ^{2}) = [(resistance in experimental hole- resistance blank hole)] x (4.67 cm 2) (membrane surface area)		219.9	244.7

Table S1. The resistance value of BBB before and after NIR irradiation.
